# Evaluation of Factors Associated With Unmet Needs in Adult Cancer Survivors in Canada

**DOI:** 10.1001/jamanetworkopen.2020.0506

**Published:** 2020-03-06

**Authors:** Saad Shakeel, Jasmine Tung, Rami Rahal, Christian Finley

**Affiliations:** 1 School of Medicine, University of Toronto, Toronto, Ontario, Canada; 2 Canadian Partnership Against Cancer, Toronto, Ontario, Canada; 3 Department of Surgery, McMaster University, Hamilton, Ontario, Canada; 4 Department of Oncology, McMaster University, Hamilton, Ontario, Canada

## Abstract

**Importance:**

Understanding the challenges faced by an increasing number of cancer survivors can guide the development and implementation of effective survivorship care models.

**Objective:**

To identify the physical, emotional, and practical concerns and associated unmet needs reported by cancer survivors.

**Design, Setting, and Participants:**

This cross-sectional survey study obtained data from the Experiences of Cancer Patients in Transitions Study of the Canadian Partnership Against Cancer, in collaboration with cancer agencies in the 10 Canadian provinces, that was disseminated in 2016. This analysis included only adult survivors aged 30 years or older who underwent chemotherapy, radiation therapy, surgical treatment, or a combination of these therapies for breast, prostate, colorectal, melanoma, or hematological cancer within the past 1 to 3 years. Data synthesis and quality assessment were conducted in 2017. Data analysis was completed in July 2019.

**Main Outcomes and Measures:**

The outcomes were the (1) quantification of the magnitude and multiplicity of the physical, emotional, and practical concerns of adult survivors of breast, colorectal, prostate, melanoma, or hematological cancer; (2) exploration of the magnitude of associated unmet needs; and (3) identification of patient-, treatment-, and cancer-specific factors associated with the reporting of unmet needs.

**Results:**

Overall, 10 717 adult respondents were included (5660 [53%] female and 6367 [60%] aged ≥65 years). The median number of concerns per respondent was 6 (interquartile range [IQR], 3-10). Among respondents with concerns, help was sought for a median of 2 (IQR, 0-4) concerns. Unmet needs were reported for a median of 4 (IQR, 2-7) concerns. Emotional concerns were reported by 8330 respondents (78%), physical concerns by 9236 respondents (86%), and practical concerns by 4668 respondents (44%). At least 1 unmet need was reported by 7033 survivors (84%) with emotional concerns, 7475 (81%) with physical concerns, and 3459 (74%) with practical concerns. Age, sex, annual income, marital status, geographic location, language, and treatment type were significant factors associated with unmet needs. Survivors of melanoma cancer had a significantly higher likelihood of reporting unmet emotional needs (odds ratio [OR], 1.75; 95% CI, 1.17-2.61; *P* = .01), whereas survivors of prostate (OR, 0.60; 95% CI, 0.43-0.84; *P* < .001) and hematological (OR, 0.70; 95% CI, 0.50-0.99; *P* = .04) cancers were significantly less likely to report unmet needs for physical concerns when compared with breast cancer survivors. Involvement of the general practitioner combined with the oncologist in providing care was associated with a significantly lower likelihood of reporting unmet emotional (OR, 0.78; 95% CI, 0.62-1.00; *P* = .05) and practical (OR, 0.72; 95% CI, 0.55-0.94; *P* = .01) needs.

**Conclusions and Relevance:**

The extent of unmet needs among cancer survivors found in this study suggests the need for enhancements in survivorship care, including better awareness of the realities of survivorship, earlier interventions for emerging concerns among survivors, and greater integration of cancer programs and primary care for more seamless transitions.

## Key Points

**Question:**

Are the health care needs of cancer survivors in Canada being met after the end of their treatment?

**Findings:**

In this cross-sectional survey study of 10 717 adult cancer survivors who participated in the Experiences of Cancer Patients in Transitions Study, almost 8 of 10 respondents who reported health care concerns across emotional, physical, and practical domains also reported an inadequate level of support, with minimal differences across cancer types. Survivors also faced a median of 6 concerns concurrently, but help was not sought or was not available for a median of 4 concerns.

**Meaning:**

Findings from this study suggest that a high level of unmet need exists among a growing number of cancer survivors, highlighting the need to enhance or implement survivorship care models that involve integrated care and a smoother transition from cancer treatment.

## Teaser

This cross-sectional survey study analyzes findings from a nationwide survey of adult cancer survivors in Canada, including the physical, emotional, and practical concerns and associated unmet needs 1 year or more after the end of treatment.

## Introduction

The Canadian health care system is facing a critical challenge: how do we provide comprehensive follow-up care that meets the individual needs of the growing number of cancer survivors in an often-siloed system? Currently, more than 1.6 million Canadian individuals are living after cancer treatment.^1^ Survival is improving for most cancers, primarily owing to early diagnosis, better staging, multimodal therapies, and improved surgical techniques. Evidence indicates the presence of physical, psychosocial, practical, and emotional well-being challenges (eg, fear of recurrence, living with uncertainty, adjusting to a new normal, and complicated comorbidities) as patients transition from treatment to the posttreatment phase of their cancer trajectory.^2,3,4,5,6,7,8,9,10^ Without appropriate support systems in place, these cancer survivors face a substantial burden because of the inadequacy of health care services in meeting the broad spectrum of needs that affect their health and quality of life.^2,11,12,13,14,15,16,17,18^

However, existing literature is limited, preventing a comprehensive understanding of the extent of unmet physical, emotional, and practical needs of cancer survivors at a national level. Moreover, existing survey tools do not capture the full extent of unmet needs, lacking comprehensiveness, internal consistency, content and structural validity, and a conceptual framework in the development process.^19^ To address this gap in knowledge, the Canadian Partnership Against Cancer, in collaboration with the 10 provincial cancer agencies, developed and disseminated the national Experiences of Cancer Patients in Transitions Study. The Canadian Partnership Against Cancer is an independent organization funded by the Canadian federal government with the long-term objectives of reducing the incidence of cancer, reducing the likelihood of dying from cancer, and improving the quality of life of those affected by cancer.

In the present study, we analyzed the Experiences of Cancer Patients in Transitions Study (referred to hereafter as survey) to identify the physical, emotional, and practical challenges encountered by adult cancer survivors between 1 and 3 years after completing curative-intent treatment. Knowledge of the gaps in care can guide the development and implementation of programs and services to address the broad scope of needs of cancer survivors.

## Methods

### Survey Development and Dissemination

Ethics and privacy approvals for the survey were obtained by the cancer agencies in the 10 Canadian provinces from their respective ethics boards before data collection. Ethics approval for this analysis was obtained from the McMaster Research Ethics Board. Written informed consent was obtained from participants in Ontario and Quebec only. In the remaining provinces, completion of the questionnaire indicated informed consent. The present study followed the American Association for Public Opinion Research (AAPOR) reporting guidelines.

The survey was developed through an iterative process based on a conceptual framework guided by literature reviews, consultations with stakeholders (ie, cancer survivors, clinicians, and system leaders), and extrapolation of existing surveys. The survey was designed to (1) assess the emotional, physical, and practical needs of cancer survivors between 1 and 3 years after treatment; (2) identify the specific needs of most survivors; (3) identify the most vulnerable survivors, along with factors associated with unmet needs; and (4) ascertain the factors and/or resources associated with needs being met. A thorough description of the survey methods, development process, pilot testing, and dissemination is published elsewhere.^20^ Twenty different concerns (listed in Table 1; eAppendix in the Supplement) were clustered into broader physical, emotional, and practical domains. The survey inquired about the severity of concern (response options: big, moderate, small, or not a concern), whether help was sought (yes or no), and how easy it was to get help for the concern (very easy, easy, hard, very hard, or didn’t get any help). Excerpts from the survey pertaining to physical, emotional, and practical domains are included in eAppendix in the Supplement; the survey is available on the Canadian Partnership Against Cancer System Performance website.

**Table 1.** Magnitude of Reported Concerns and Unmet Needs Across Physical, Emotional, and Practical Domains

Domain	No. of Responses	No. (%)		
		Concern Reported	Help Sought	Unmet Need Reported
Physical concerns				
Swelling	10 322	2411 (23)	1499 (64)	958 (40)
Fatigue	10 555	7210 (68)	2573 (38)	4702 (65)
Hormonal menopause	10 227	2663 (26)	1300 (51)	1417 (53)
Chronic pain	10 375	3573 (34)	2094 (61)	1530 (43)
Bladder incontinence	10 475	3716 (36)	2050 (58)	1650 (44)
Gastrointestinal tract problems	10 432	3824 (37)	2304 (63)	1521 (40)
Nervous system problems	10 413	3889 (37)	1967 (53)	2123 (55)
Change in concentration or memory	10 426	4108 (39)	1016 (26)	3093 (75)
Change in sexual activity	10 513	4748 (45)	1789 (39)	3027 (64)
Emotional concerns				
Depression	9700	4527 (47)	1459 (33)	3010 (67)
Anxiety	9925	6815 (69)	1996 (31)	4641 (68)
Change in relationship with family	10 632	3448 (32)	674 (21)	2679 (78)
Change in relationship with friends	10 577	2235 (21)	270 (13)	1851 (83)
Change in body image	10 594	4184 (40)	849 (22)	3152 (75)
Change in sexual intimacy	10 551	4488 (43)	1282 (30)	3151 (70)
Practical concerns				
Returning to work or school	10 388	2364 (23)	736 (32)	1625 (69)
Getting to and from appointments	10 521	2240 (21)	894 (43)	1229 (55)
Taking care of family	10 399	1349 (13)	367 (29)	924 (69)
Difficulty getting health or life insurance	10 364	1642 (16)	457 (30)	1192 (73)
Paying health care bills	10 469	2116 (20)	696 (36)	1373 (65)

The survey was disseminated in 2016 to eligible participants through the mail but provided recipients with the option of completing it online. The survey package included a cover letter, with a description of the study, confidentiality agreement, URL link and personal identification number to the encrypted online survey, and informed consent documents in Ontario and Quebec (in which written consent was required, whereas completion of the questionnaire indicated consent in the other provinces); the paper survey with a preprinted barcode and associated personal identification number (for those who preferred to complete the paper version); and a preaddressed, prepaid return envelope. Designated staff were available to assist respondents during the data collection phase of the study.

The study population was selected through probability sampling. A response rate of 30% was assumed, and thus the adult sample population was calculated with a margin of sampling error of ±5% at the 95% CI by disease site and province.^20^ The number of eligible survivors for each province was estimated from the prevalence of specific cancer type at the national level and the incidence at the provincial level. The smaller provinces were less likely to achieve adequate sample size, owing to CI precision. Therefore, all eligible survivors were surveyed in smaller provinces. In larger provinces, the number of eligible participants was larger than that required according to the sample size calculation; therefore, random samples for disease sites were drawn. Furthermore, an external vendor was hired to assist with survey distribution, design, and preliminary analysis. Therefore, confidential data on survivor characteristics could not be shared with the vendor, which was required to assign weighting to the survey to make the sample representative of all Canadian cancer survivors. Thus, this sample should not be generalized to represent all survivors in Canada.

### Study Population

The original study population consisted of adolescent and adult patients with cancer who completed treatment within the past 1 to 3 years (n = 40 790). Adult participants aged 30 years or older included survivors of primary nonmetastatic breast, colorectal, prostate, melanoma, or hematological (eg, Hodgkin lymphoma, diffuse B-cell lymphoma, acute myelogenous leukemia, and acute lymphocytic lymphocyte leukemia) cancer. Participants aged 15 to 29 years included survivors of any nonmetastatic cancer, except testicular cancer that included metastatic cases. In this study, we included only those survivors aged 30 years or older who received chemotherapy, radiation therapy, surgical treatment, or a combination of these therapies.

Cancer survivors were identified by the provincial cancer agencies using provincial cancer registries in 2016. Those who resided in the Canadian territories were not included in this study. A total of 40 790 survey packages were mailed across the 10 provinces and 13 319 responses were received (response rate = 33%); 12 929 surveys were completed by survivors aged 30 years or older. Responses were included in this analysis if the respondents completed all survey questions about disease site and type of treatment received as well as about physical, emotional, and practical concerns, help sought, and ease of getting help. Respondents who received treatment for multiple cancers were excluded given the complexity of their health care needs.

### Outcomes

The outcomes of this study were as follows: (1) quantification of the magnitude (proportion) and multiplicity (median) of the physical, emotional, and practical concerns reported by adult survivors of breast, colorectal, prostate, melanoma, or hematological cancer; (2) exploration of the magnitude (proportion) of associated unmet needs; and (3) identification of patient-, treatment-, clinician-, and cancer-specific factors associated with the reporting of unmet needs. *Unmet need* was defined as the percentage of respondents who did not receive help regardless of whether they sought help.

### Statistical Analysis

The binary variables for physical, emotional, and practical domains and subsequent unmet needs were derived by aggregating the responses to individual concerns under these 3 domains. These variables were used to identify the extent of concerns and associated unmet needs. For instance, if the participants reported any concern (of low, moderate, or high severity) under emotional domain, the emotional concern variable was coded as yes. Subsequently, the unmet need for the emotional concern variable was coded as yes if the respondents did not seek any help, sought help for a particular concern but reported that it was hard or very hard to get help, or did not get any help despite seeking it (eAppendix in the Supplement includes relevant excerpts from the survey).

Univariate analysis for binary and categorical variables was performed using the χ^2^ test to identify the important factors for inclusion in the regression models. Multivariate logistic regression analysis was performed to determine the adjusted associations between reporting of unmet needs in emotional, physical, and practical domains and independent variables. Using a forward stepwise approach, we sequentially included in the models the patient demographic-, treatment-, clinician-, and site-specific *P* values. A 2-sided *P* < .05 was considered to be statistically significant.

All analyses were performed with Stata, version 9.3 (StataCorp LLC). Data synthesis and quality assessment were conducted in 2017. Data analysis was completed by independent researchers in July 2019.

## Results

In total, 10 717 adult respondents were included in this analysis. Among these respondents, 5660 (53%) were female and 6367 (60%) were aged 65 years or older. Most respondents (6405 [60%]) were retired, and 2736 of 8318 respondents (33%) had an annual income of Canadian $75 000 (approximately US $56 906) or more.

The respondents comprised 3607 survivors (34%) of breast cancer, 2607 (24%) of prostate cancer, 2259 (21%) of colorectal cancer, 981 (9%) of hematological cancer, and 1265 (12%) of melanoma. Table 2 presents the distribution of demographic and treatment-specific factors by disease type.

**Table 2.** Distribution of Demographic-, Cancer-, and Treatment-Specific Factors by Cancer Type

Variable	No. (%)				
	Breast	Prostate	Colorectal	Hematological	Melanoma
Sample size, No.	3607	2607	2259	981	1265
Age group, y					
30-44	181 (5)	<5	25 (1)	140 (14)	110 (9)
45-54	588 (16)	78 (3)	160 (7)	122 (12)	161 (13)
55-64	1062 (29)	555 (21)	569 (25)	241 (25)	356 (28)
65-74	1136 (32)	1270 (49)	820 (36)	263 (27)	371 (29)
≥75	638 (18)	702 (27)	685 (30)	215 (22)	267 (21)
Sex					
Male	11 (0)	2601 (100)	1255 (56)	525 (54)	635 (50)
Female	3587 (100)	NA	995 (44)	453 (46)	625 (50)
Chronic condition					
No	1268 (36)	897 (34)	704 (32)	403 (43)	447 (37)
Yes	2214 (64)	1669 (66)	1473 (68)	542 (57)	774 (63)
Income, Can$^a^					
<25 000	470 (18)	244 (12)	347 (20)	144 (18)	96 (9)
25 000-50 000	758 (29)	683 (32)	565 (32)	232 (29)	249 (24)
>50 000-75 000	560 (21)	496 (24)	345 (20)	163 (21)	230 (23)
>75 000-125 000	493 (19)	430 (20)	298 (17)	149 (19)	238 (23)
>125 000	377 (14)	253 (12)	190 (11)	101 (13)	207 (20)
Immigration					
No	2992 (84)	2150 (83)	1821 (82)	815 (84)	1098 (88)
Yes	558 (16)	418 (16)	412 (19)	154 (16)	155 (12)
Marital status					
Single	216 (6)	108 (4)	132 (6)	71 (7)	78 (6)
Married or partnered	2531 (71)	2172 (84)	1623 (72)	732 (76)	972 (77)
Divorced, separated, or widowed	832 (23)	308 (12)	492 (22)	166 (17)	209 (17)
Language					
English	2984 (84)	2172 (85)	1858 (83)	599 (62)	985 (79)
French	447 (13)	333 (13)	281 (13)	334 (35)	248 (20)
Other	123 (3)	63 (3)	91 (4)	35 (4)	18 (1)
Educational level					
≤High school diploma	519 (15)	585 (23)	480 (22)	177 (19)	136 (11)
<College	1177 (34)	689 (27)	711 (32)	312 (33)	356 (29)
Some university	938 (27)	573 (23)	551 (25)	203 (21)	320 (26)
Bachelor’s, master’s, or doctorate degree	864 (25)	690 (27)	454 (21)	256 (27)	431 (35)
Employment status					
Full-time	782 (22)	457 (18)	390 (18)	248 (26)	357 (29)
Part-time	403 (12)	202 (8)	165 (8)	73 (8)	94 (8)
Other	398 (11)	84 (3)	171 (8)	150 (16)	78 (6)
Retired	1921 (55)	1818 (71)	1473 (67)	484 (51)	709 (57)
Population size of geographic location					
<2000	791 (22)	649 (25)	501 (23)	167 (17)	249 (20)
2000-10 000	457 (13)	372 (14)	339 (15)	122 (13)	160 (13)
>10 000-50 000	627 (18)	394 (15)	377 (17)	196 (20)	245 (20)
>50 000	1652 (47)	1154 (45)	1001 (45)	478 (50)	592 (48)
Treatment type					
Surgical treatment only	275 (8)	813 (31)	1049 (46)	10 (1)	1107 (88)
Chemotherapy only	116 (3)	101 (4)	100 (4)	578 (59)	27 (2)
Radiation therapy only	223 (6)	965 (37)	21 (1)	10 (1)	17 (1)
Surgical treatment + radiation therapy	541 (15)	46 (2)	567 (25)	71 (7)	57 (5)
Surgical treatment + chemotherapy	513 (14)	184 (7)	71 (3)	7 (1)	27 (2)
Surgical treatment + chemoradiation	1777 (49)	104 (4)	415 (18)	59 (6)	21 (2)
Chemoradiation	160 (4)	394 (15)	36 (2)	246 (25)	9 (1)
GP involvement					
None	1004 (29)	851 (34)	646 (30)	355 (39)	555 (47)
Somewhat	1214 (35)	902 (36)	695 (33)	266 (29)	333 (28)
Very involved	1246 (36)	738 (30)	791 (37)	287 (32)	294 (25)
Physician type					
GP	1155 (33)	388 (15)	534 (25)	53 (6)	259 (23)
Oncologist	1099 (32)	1297 (52)	859 (40)	634 (66)	577 (51)
Both	1234 (35)	829 (33)	763 (35)	278 (29)	290 (26)
Emotional concerns	2912 (81)	2130 (83)	1605 (72)	795 (82)	888 (71)
Unmet emotional needs	2469 (85)	1776 (83)	1365 (85)	664 (84)	759 (86)
Emotional concerns, median (IQR)	2 (1-4)	2 (1-4)	2 (0-3)	3 (1-4)	1 (0-3)
Unmet emotional needs, median (IQR)	2 (1-3)	2 (1-3)	2 (1-3)	2 (1-4)	1 (1-3)
Physical concerns	3221 (91)	2464 (96)	1942 (88)	925 (96)	684 (56)
Unmet physical needs	2747 (85)	1896 (77)	1557 (80)	745 (81)	530 (78)
Physical concerns, median (IQR)	4 (2-6)	3 (2-5)	3 (1-5)	4 (2-6)	1 (0-3)
Unmet physical needs, median (IQR)	2 (1-4)	2 (1-3)	2 (1-3)	2 (1-3)	2 (1-2)
Practical concerns	1767 (50)	943 (37)	953 (44)	542 (57)	463 (37)
Unmet practical needs	1275 (72)	728 (77)	703 (74)	391 (72)	362 (78)
Practical concerns, median (IQR)	1 (0-2)	0 (0-1)	0 (0-1)	1 (0-2)	0 (0-1)
Unmet practical needs, median (IQR)	1 (1-2)	1 (1-2)	1 (1-2)	1 (1-2)	1 (1-2)

Abbreviations: Can$, Canadian dollar; GP, general practitioner; IQR, interquartile range; NA, not applicable.

^a^ To convert Can$ to US dollars, multiply by 0.75.

The median (interquartile range [IQR]) number of concerns per respondent was 6 (3-10). Among those with concerns, help was sought for a median (IQR) of 2 (0-4) concerns. Unmet needs (whether no help was sought, help was sought but was hard or very hard to get, or no help was received despite seeking it) were reported for a median (IQR) of 4 (2-7) concerns. Emotional concerns were reported by 8330 respondents (78%), physical concerns by 9236 respondents (86%), and practical concerns by 4668 respondents (44%). At least 1 unmet need was reported by 7033 (84%) of those with emotional concerns, 7475 (81%) of those with physical concerns, and 3459 (74%) of those with practical concerns.

The extent of reported emotional concerns ranged from 71% of melanoma cancer survivors (n = 888) to 83% of prostate cancer survivors (n = 2130); likewise, physical concerns ranged from 56% of melanoma cancer survivors (n = 684) to 96% of hematological cancer survivors (n = 925), and practical concerns ranged from 37% of melanoma cancer survivors (n = 463) to 57% of hematological cancer survivors (n = 542). Subsequently, the extent of unmet needs for emotional concerns was reported by more than 83% of prostate cancer survivors (n = 1776) to 86% of melanoma cancer survivors (n = 759). The proportion of survivors who reported unmet needs for physical concerns ranged from 77% of prostate cancer survivors (n = 1896) to 85% of breast cancer survivors (n = 2747). Unmet needs for practical concerns ranged from 72% of breast (n = 1275) and hematological (n = 391) cancer survivors to 78% of melanoma cancer survivors (n = 362).

### Types of Concerns and Unmet Needs

Table 1 presents the magnitude of different concerns reported and their associated unmet needs. Fatigue (reported by 7210 survivors [68%]), change in sexual activity (4748 [45%]), change in concentration and memory (4108 [39%]), and nervous system problems (3889 [37%]) were the top reported physical concerns with the highest proportion of associated unmet needs: 65% (n = 4702) experienced fatigue, 64% (n = 3027) experienced a change in sexual activity, 75% (n = 3093) experienced a change in concentration and memory, and 55% (n = 2123) experienced nerve problems. Anxiety (reported by 6815 survivors [69%]), depression (4527 [47%]), change in sexual intimacy (4488 [43%]), and change in body image (4184 [40%]) were at the top of reported emotional concerns; however, more than 70% of the respondents reported unmet needs regardless of the type of emotional concern. In contrast, less than 25% of the respondents reported any type of practical concern. The extent of unmet practical needs ranged from getting to and from appointments (reported by 1229 survivors [55%]) to difficulty getting health or life insurance (1192 [73%]).

### Severity of Concerns and Unmet Needs

Overall, 4094 respondents (38%) reported concerns in all 3 domains, 4106 (38%) in 2 domains, and 1740 (16%) in 1 domain (Figure). No concerns were reported by 777 respondents (7%). Among those with concerns in 2 domains, 3609 (88%) reported emotional and physical concerns concurrently. Of those with concern in only 1 domain, 1212 (70%) reported concerns with physical health.

**Figure.**
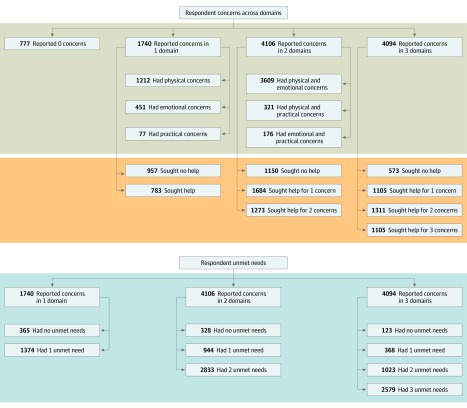
Distribution of Respondents Who Reported Concerns, Help Sought, and Unmet Needs

Most participants reported having their needs unmet. Among those with concerns in 3 domains, 2579 (63%) reported unmet needs across all 3 domains; of those with concerns in 2 domains, 2833 (69%) had unmet needs in both domains; and of those with concerns in 1 domain, 1374 (79%) had an unmet need in that domain (Figure). Help for all of their concerns was sought by 1105 participants (27%) with 3 concerns in all 3 domains, by 1273 (31%) of those with 2 concerns in 2 domains, and by 783 (45%) of those with concern in 1 domain (Figure).

### Factors Associated With Unmet Needs

The results of multivariate analyses are summarized in Table 3. Survivors who received chemoradiation alone had a significantly higher likelihood of reporting unmet emotional (odds ratio [OR], 1.54; 95% CI, 1.17-2.01; *P* = <.001) and physical (OR, 1.46; 95% CI, 1.17-1.81; *P* < .001) needs, compared with those who received surgical treatment alone. Similarly, those who received chemoradiation after surgical treatment also had a significantly higher likelihood of reporting unmet emotional (OR, 1.69; 95% CI, 1.31-2.18; *P* < .001) and physical (OR, 1.78; 95% CI, 1.44-2.21; *P* < .001) needs compared with those who received surgical treatment alone.

**Table 3.** Results of Multivariate Analyses

Variable	Emotional Unmet Needs		Physical Unmet Needs		Practical Unmet Needs	
	OR (95% CI)	*P* Value	OR (95% CI)	*P* Value	OR (95% CI)	*P* Value
Age group (reference: <55), y						
55-64	0.84 (0.64-1.11)	.22	0.97 (0.75-1.25)	.80	0.81 (0.61-1.08)	.15
65-74	0.81 (0.58-1.14)	.24	0.99 (0.73-1.34)	.94	0.71 (0.49-1.03)	.07
≥75	1.02 (0.68-1.55)	.91	1.15 (0.81-1.65)	.43	0.48 (0.30-0.75)	<.001
Female sex (reference: male)	0.89 (0.67-1.18)	.40	0.99 (0.78-1.26)	.97	0.74 (0.55-1.00)	.05
Chronic condition	0.90 (0.74-1.08)	.25	0.99 (0.84-1.16)	.90	0.88 (0.72-1.09)	.25
Treatment (reference: surgical treatment)						
Chemotherapy or radiation therapy only	1.54 (1.17-2.01)	<.001	1.46 (1.17-1.81)	<.001	1.01 (0.71-1.44)	.95
Surgical treatment +/− chemotherapy +/− radiation therapy	1.69 (1.31-2.18)	<.001	1.78 (1.44-2.21)	<.001	0.81 (0.60-1.10)	.18
Physician type (reference: family physician)						
Oncologist	0.73 (0.57-0.94)	.02	0.90 (0.73-1.12)	.35	0.81 (0.61-1.07)	.14
Family physician + oncologist	0.78 (0.62-1.00)	.05	0.89 (0.72-1.10)	.27	0.72 (0.55-0.94)	.01
Family physician involvement (reference: no)						
Somewhat involved	0.97 (0.77-1.21)	.76	0.91 (0.75-1.10)	.32	0.99 (0.78-1.26)	.92
Very involved	0.60 (0.47-0.75)	<.001	0.62 (0.51-0.76)	<.001	0.75 (0.58-0.97)	.03
Income (reference: <25 000), Can$^a^						
25 000-50 000	1.01 (0.75-1.36)	.96	1.14 (0.87-1.48)	.34	0.89 (0.65-1.22)	.48
>50 000-75 000	1.18 (0.84-1.66)	.35	1.20 (0.90-1.61)	.22	1.10 (0.76-1.59)	.62
>75 000-125 000	0.83 (0.58-1.18)	.30	1.06 (0.78-1.45)	.70	0.65 (0.45-0.96)	.03
>125 000	0.83 (0.55-1.23)	.35	0.94 (0.66-1.33)	.72	0.55 (0.35-0.84)	.01
Immigration (reference: no)	0.99 (0.76-1.29)	.95	0.96 (0.77-1.20)	.74	1.31 (0.97-1.77)	.08
Marital status (reference: single)						
Married or partnered	1.30 (0.90-1.88)	.16	0.90 (0.64-1.26)	.54	1.77 (1.21-2.58)	<.001
Divorced or widowed	1.07 (0.72-1.59)	.72	0.86 (0.60-1.25)	.43	1.10 (0.74-1.64)	.64
Language (reference: English)						
French	1.31 (1.01-1.71)	.05	1.29 (1.02-1.62)	.03	1.41 (1.05-1.89)	.02
Other	0.64 (0.38-1.07)	.09	1.18 (0.73-1.92)	.50	0.93 (0.51-1.69)	.81
Educational level (reference: <high school diploma)						
<College	0.89 (0.65-1.20)	.43	1.02 (0.79-1.32)	.90	1.09 (0.77-1.54)	.62
Some university	1.00 (0.73-1.39)	.98	0.99 (0.76-1.30)	.97	1.04 (0.73-1.48)	.84
Bachelor’s, master’s, or doctorate degree	0.90 (0.65-1.25)	.53	0.78 (0.59-1.03)	.09	0.89 (0.61-1.30)	.55
Employment status (reference: full-time)						
Part-time	0.86 (0.63-1.18)	.34	1.09 (0.80-1.48)	.60	1.31 (0.90-1.91)	.16
Other	0.94 (0.67-1.31)	.70	0.75 (0.56-1.02)	.06	0.73 (0.53-1.01)	.06
Retired	1.05 (0.80-1.39)	.72	0.85 (0.67-1.09)	.21	0.73 (0.53-1.00)	.05
Population size of geographic location (reference: <2000 individuals)						
2000-10 000	0.93 (0.69-1.26)	.65	1.02 (0.78-1.34)	.86	1.04 (0.74-1.48)	.81
>10 000-50 000	0.95 (0.72-1.27)	.74	0.90 (0.70-1.15)	.41	0.76 (0.56-1.05)	.09
>50 000	0.88 (0.70-1.12)	.30	0.76 (0.62-0.93)	.01	0.77 (0.59-1.00)	.05
Internet use (reference: daily to once/mo)						
Never	0.92 (0.67-1.27)	.62	1.10 (0.84-1.44)	.51	0.88 (0.62-1.26)	.50
Social media use (reference: daily to once/mo)						
Never	1.08 (0.88-1.31)	.46	1.15 (0.97-1.36)	.11	1.10 (0.87-1.38)	.43
Cancer type (reference: breast)						
Prostate	0.99 (0.67-1.46)	.96	0.60 (0.43-0.84)	<.001	1.29 (0.83-1.99)	.26
Colorectal	1.28 (0.94-1.75)	.12	0.81 (0.62-1.06)	.13	1.14 (0.83-1.56)	.42
Hematological	0.98 (0.66-1.46)	.91	0.70 (0.50-0.99)	.04	0.90 (0.59-1.35)	.60
Melanoma	1.75 (1.17-2.61)	.01	0.84 (0.59-1.21)	.35	1.34 (0.85-2.11)	.21

Abbreviations: Can$, Canadian dollar; OR, odds ratio.

^a^ To convert Can$ to US dollars, multiply by 0.75.

When general practitioners were very involved with survivorship care, the respondents reported a significantly lower likelihood of having unmet emotional (OR, 0.60; 95% CI, 0.47-0.75; *P* < .001), physical (OR, 0.62; 95% CI, 0.51-0.76; *P* < .001), and practical (OR, 0.75; 95% CI, 0.58-0.97; *P* = .01) needs. In addition, significantly less reporting of unmet emotional (OR, 0.78; 95% CI, 0.62-1.00; *P* = .05) and practical (OR, 0.72; 95% CI, 0.55-0.94; *P* = .03) needs occurred when both the general practitioner and the oncologist were involved in care, but not for physical concerns.

With regard to patient-specific factors, female survivors had significantly lower likelihood of reported unmet practical needs (OR, 0.74; 95% CI, 0.55-1.00; *P* = .05). Retired survivors (OR, 0.73; 95% CI, 0.53-1.00; *P* = .05) with an annual income between Canadian $75 000 and $125 000 (OR, 0.65; 95% CI, 0.45-0.96; *P* = .03) or greater than $125 000 (OR, 0.55; 95% CI, 0.35-0.84; *P* = .01) reported significantly fewer unmet practical needs, whereas married or partnered respondents (OR, 1.77; 95% CI, 1.21-2.58; *P* < .001) reported significantly more unmet practical needs. Survivors in urban areas (locations with >50 000 residents) had a significantly lower likelihood of reporting unmet physical (OR, 0.76; 95% CI, 0.62-0.93; *P* = .01) and practical (OR, 0.77; 95% CI, 0.59-1.00; *P* = .05) needs.

French-speaking Canadian respondents had a significantly higher likelihood of reporting unmet needs across emotional (OR, 1.31; 95% CI, 1.01-1.71; *P* = .05), physical (OR, 1.29; 95% CI, 1.02-1.62; *P* = .03), and practical (OR, 1.41; 95% CI, 1.05-1.89; *P* = .02) domains.

Survivors of breast cancer had the largest sample size (n = 3607) and reported one of the highest proportions of unmet needs across all 3 domains in unadjusted analysis (Table 2). Therefore, breast cancer was used as a reference group in the adjusted analysis to identify the differences in unmet needs by cancer type. When compared with breast cancer survivors, melanoma cancer survivors had a significantly higher likelihood of reporting unmet emotional needs (OR, 1.75; 95% CI, 1.17-2.61; *P* = .01), whereas survivors of prostate (OR, 0.60; 95% CI, 0.43-0.84; *P* < .001) and hematological (OR, 0.70; 95% CI, 0.50-0.99; *P* = .04) cancers were significantly less likely to report unmet needs for physical concerns. No differences in reporting of unmet practical needs were found between cancer types.

## Discussion

To our knowledge, the Experiences of Cancer Patients in Transitions Study is the largest pan-Canadian survey of its kind that captures the experiences and broad needs of cancer survivors 1 to 3 years after completion of their treatment. Findings from this survey highlighted that most survivors faced physical, emotional, and practical concerns after completing treatment and that a high proportion of them did not seek help or receive adequate support for their concerns. Of those who did not seek help, a large proportion indicated they did not know help was available, or they were told that nothing could be done for their problems.^20^ The data also suggested that the concerns spanned multiple domains concurrently, emphasizing the broad spectrum of support required to address those needs. Given the considerable amount of unmet needs of survivors observed in this study, it appears that enhancements to survivorship or follow-up care are needed, including increasing awareness of the realities of survivorship, implementing earlier interventions for emerging concerns among survivors, and improving the integration of cancer programs and primary care for a smoother transition from cancer to survivorship care.

The observed prevalence of concerns among survivors was high in this study, compared with that in other published studies.^10,21,22,23,24,25,26,27^ Harrison et al^2^ reviewed 14 studies (sample size of 69-913) on unmet needs of survivors of breast, prostate, gynecological, or melanoma cancer and found a lower range of unmet physical (26%-52% vs 89%), psychosocial (8%-17% vs 86%), and economic (5%-13% vs 82%) needs, compared with the ranges in this study. Possible explanations for this stark difference could be that the survey incorporated some of the lesser reported concerns, in addition to having a larger nationally representative sample of survivors. For instance, although fatigue, cognitive effects, pain, anxiety, and depression have been reported frequently, neuropathy as well as changes in sexuality, sexual intimacy, body image, and relationships have been less frequently included and acknowledged in studies.^21,22,23,25,27^ The national Livestrong surveys in the United States reported comparable overall prevalence of physical, emotional, and practical concerns; however, the Livestrong surveys found considerably lower levels of unmet emotional (50% vs 86%) and physical (42% vs 89%) needs compared with levels in the present study.^28^ This difference is likely due to the dissimilarity of the survivors in each study. The Livestrong surveys included survivors of breast, colorectal, and prostate cancers who were white, well educated, and well insured, with high economic security. The Experiences of Cancer Patients in Transitions Study comprised survivors of 5 cancer sites with equal representation of annual income, geographic location, and educational status. Although we did not compare unmet needs across the different health care systems, we anticipate broader health care structure, barriers in access to care, and variations in funding models to have some implications for the extent of unmet needs. We believe further research is needed into the differences in challenges faced by cancer survivors according to health care structures and funding models.

With regard to factors associated with survivors reporting positive outcomes, our analysis showed that surgical therapy alone, older age, higher income, residency in an urban area (>50 000 population), fluency in English, and involvement of both a general practitioner and an oncologist were associated with a higher likelihood that the survivors’ needs were met. When compared with breast cancer survivors, survivors of melanoma cancer had a significantly higher likelihood of experiencing unmet emotional needs, whereas prostate and hematological cancer survivors had a significantly lower likelihood of experiencing unmet physical needs. These findings suggest that survivors of certain types of cancer may be at an increased risk of experiencing challenges during survivorship.

These findings can be used to develop tools for risk stratification (based on demographic-, treatment-, and disease-specific factors) of patients with cancer before they transition to the survivorship phase. Setting up appropriate resources for these high-risk patients before the survivorship phase could help reduce the burden and severity of their unmet needs and thus improve their quality of life. More research is needed to develop needs assessments and risk stratification tools that will assist care practitioners with prescribing appropriate follow-up care pathways and an appropriate level of support for current and future health care needs.

Many cancer survivors require follow-up care to manage the effects of cancer and the treatment received and to coordinate the care for all survivorship issues.^4^ The findings of this study confirmed the advantage of involving oncologists and primary care physicians in patient care, which has been associated with positive outcomes during survivorship.^29^ However, even in the publicly funded health care system in Canada, patients do not always have access to a general practitioner, the oncologist, or both after completion of cancer treatment. Furthermore, there appears to be a need for the development of a unified, broad, and holistic survivorship care model that aims to provide equitable access to survivors across Canada. The primary care sector and the cancer system often function in relative isolation from one another, resulting in challenges in information flow (eg, among care practitioners and between care practitioners and patients) and continuity of care. For survivors, this situation means not knowing what to expect once treatment is complete, feeling unsure about who is responsible for their follow-up care, or losing needed supports at the conclusion of treatment.^30^ Despite the widespread acknowledgment of the importance of integrated survivorship care, the optimal survivorship care model that clarifies practitioner roles and responsibilities remains poorly understood, and implementation of efforts remains fragmented.^31^ We suggest that such a model be tailored to address barriers, including funding constraints, staffing shortages, and lack of shared information systems between different health care sectors.^31^

The actionable recommendations from these findings are as follows: (1) better patient education, to teach patients to recognize health care concerns that are not a normal part of cancer survivorship; (2) early involvement of primary care physicians in cancer care, to ensure the smooth transition from cancer treatment to survivorship care overseen by a primary care physician; (3) improved coordination and communication between primary care physicians and oncologists, to provide holistic support to survivors; (4) access to alternative care practitioners (eg, nurses, occupational therapists, and social workers) in geographic areas with a shortage of primary care physicians; and (5) development of tools for risk stratification of patients during cancer treatment and preemptive allocation of resources to address an anticipated high level of needs based on predisposing factors. More research is required into how communication, coordination, and integration between cancer care teams and primary care physicians can successfully occur to ensure that survivor needs are met in the right way, at the right time, and by the most appropriate professional.

### Limitations

This study has a few limitations. First, the survey tool was not validated; however, robust measures were taken to evaluate internal validity and pilot testing before the survey was disseminated. Second, the response rate (33%) and inability to apply weightings given confidentiality concerns may limit the generalizability to all cancer survivors in Canada. However, this survey was a large national study of participants selected from provincial cancer registries and hence was more generalizable than others in the literature. Third, the sample may include a large proportion of older adults with other comorbidities that may cause symptoms such as pain, mobility limitations, fatigue, and emotional distress. Thus, distinguishing the symptoms associated with cancer from those associated with other comorbidities may not have been easy for survey respondents and may have led to overreporting in some cases. However, the presence of comorbidities was not a significant factor in unmet needs in the adjusted analysis. Nonetheless, living with comorbidities is a reality for survivors and may add to the complexities of recovery and coping with the aftermath of cancer.

## Conclusions

As the number of cancer survivors continues to grow, identifying and addressing their needs is imperative to ensure the provision of high-quality survivorship care. Our findings suggest that the high prevalence of unmet needs in this population underscores the need for system-level changes, including earlier identification of patients at high risk for challenges during survivorship, models of shared care that include cancer specialists and primary care teams, and increased knowledge about the importance of providing adequate survivorship care that will enhance the quality of life for cancer survivors in Canada.

## Notes

Supplement. **eAppendix.** Excerpts From Survey Questions on Physical, Emotional, and Practical Concerns, Help Sought, and Unmet Needs
