# Supplementary material for: Evaluation of Factors Associated With Unmet Needs in Adult Cancer Survivors in Canada
Source: JAMA Netw Open. 2020 Mar 6;3(3):e200506. doi: 10.1001/jamanetworkopen.2020.0506 (PMC7060489; doi:10.1001/jamanetworkopen.2020.0506)
Supplement: Supplement. — eAppendix. Excerpts From Survey Questions on Physical, Emotional, and Practical Concerns, Help Sought, and Unmet Needs [file jamanetwopen-3-e200506-s001.pdf]

Supplementary Online Content

Shakeel S, Tung J, Rahal R, Finley C. Evaluation of factors associated with unmet needs in adult cancer survivors in Canada. *JAMA Netw Open.* 2020;3(3):e200506. doi:10.1001/jamanetworkopen.2020.0506

eAppendix. Excerpts From Survey Questions on Physical, Emotional, and Practical Concerns, Help Sought, and Unmet Needs

This supplementary material has been provided by the authors to give readers additional information about their work.

©2020 Shakeel S et al. *JAMA Network Open.*

eAppendix. Excerpts From Survey Questions on Physical, Emotional, and Practical Concerns, Help Sought, and Unmet Needs

PHYSICAL CHALLENGES

	How much was this a concern for you?	Did you seek help for this concern?	How easy was it to get help for this concern?
Swelling of arms or legs (lymphedema)	□ Big<br>□ Moderate<br>□ Small<br>□ Not a concern, **move to next item**	□ Yes<br>□ No…**Next item**	□ Very easy<br>□ Easy<br>□ Hard<br>□ Very hard<br>□ Didn't get any help
Fatigue, tiredness	□ Big<br>□ Moderate<br>□ Small<br>□ Not a concern, **move to next item**	□ Yes<br>□ No…**Next item**	□ Very easy<br>□ Easy<br>□ Hard<br>□ Very hard<br>□ Didn't get any help
Hormonal, menopause, or fertility	□ Big<br>□ Moderate<br>□ Small<br>□ Not a concern, **move to next item**	□ Yes<br>□ No…**Next item**	□ Very easy<br>□ Easy<br>□ Hard<br>□ Very hard<br>□ Didn't get any help
Chronic pain or long term pain	□ Big<br>□ Moderate<br>□ Small<br>□ Not a concern, **move to next item**	□ Yes<br>□ No…**Next item**	□ Very easy<br>□ Easy<br>□ Hard<br>□ Very hard<br>□ Didn't get any help
Bladder and/ or urinary problems (e.g., incontinence)	□ Big<br>□ Moderate<br>□ Small<br>□ Not a concern, **move to next item**	□ Yes<br>□ No…**Next item**	□ Very easy<br>□ Easy<br>□ Hard<br>□ Very hard<br>□ Didn't get any help
Gastrointestinal problems (e.g., digestion and/or bowel issues)	□ Big<br>□ Moderate<br>□ Small<br>□ Not a concern, **move to next item**	□ Yes<br>□ No…**Next item**	□ Very easy<br>□ Easy<br>□ Hard<br>□ Very hard<br>□ Didn't get any help
Nerve problems (numbness or tingling)	□ Big<br>□ Moderate<br>□ Small<br>□ Not a concern, **move to next item**	□ Yes<br>□ No…**Next item**	□ Very easy<br>□ Easy<br>□ Hard<br>□ Very hard<br>□ Didn't get any help
Changes to concentration, memory	□ Big<br>□ Moderate<br>□ Small<br>□ Not a concern, **move to next item**	□ Yes<br>□ No…**Next item**	□ Very easy<br>□ Easy<br>□ Hard<br>□ Very hard<br>□ Didn't get any help
Changes in sexual activity or function	□ Big<br>□ Moderate<br>□ Small<br>□ Not a concern, **move to next item**	□ Yes<br>□ No…**Next item**	□ Very easy<br>□ Easy<br>□ Hard<br>□ Very hard<br>□ Didn't get any help

©2020 Shakeel S et al. *JAMA Network Open.*

EMOTIONAL CHALLENGES

	**How much was this a concern for you?**	**Did you seek help for this concern?**	**How easy was it to get help for this concern?**
Depression, sadness, loss of interest in everyday things	□ Big<br>□ Moderate<br>□ Small<br>□ Not a concern, **move to next item**	□ Yes<br>□ No…**Next item**	□ Very easy<br>□ Easy<br>□ Hard<br>□ Very hard<br>□ Didn't get any help
Anxiety, stress, worry about cancer returning	□ Big<br>□ Moderate<br>□ Small<br>□ Not a concern, **move to next item**	□ Yes<br>□ No…**Next item**	□ Very easy<br>□ Easy<br>□ Hard<br>□ Very hard<br>□ Didn't get any help
Changes in relationships with family, partners	□ Big<br>□ Moderate<br>□ Small<br>□ Not a concern, **move to next item**	□ Yes<br>□ No…**Next item**	□ Very easy<br>□ Easy<br>□ Hard<br>□ Very hard<br>□ Didn't get any help
Changes in relationships with friends or coworkers	□ Big<br>□ Moderate<br>□ Small<br>□ Not a concern, **move to next item**	□ Yes<br>□ No…**Next item**	□ Very easy<br>□ Easy<br>□ Hard<br>□ Very hard<br>□ Didn't get any help
Changes in body image (i.e., confidence in appearance, etc.)	□ Big<br>□ Moderate<br>□ Small<br>□ Not a concern, **move to next item**	□ Yes<br>□ No…**Next item**	□ Very easy<br>□ Easy<br>□ Hard<br>□ Very hard<br>□ Didn't get any help
Changes in sexual intimacy	□ Big<br>□ Moderate<br>□ Small<br>□ Not a concern, **move to next item**	□ Yes<br>□ No…**Next item**	□ Very easy<br>□ Easy<br>□ Hard<br>□ Very hard<br>□ Didn't get any help

©2020 Shakeel S et al. *JAMA Network Open.*

PRACTICAL CHALLENGES

	**How much was this a concern for you?**	**Did you seek help for this concern?**	**How easy was it to get help for this concern?**
Returning to work or school, now or in the future	□ Big<br>□ Moderate<br>□ Small<br>□ Not a concern, **move to next item**	□ Yes<br>□ No…**Next item**	□ Very easy<br>□ Easy<br>□ Hard<br>□ Very hard<br>□ Didn't get any help
Getting to and from appointments	□ Big<br>□ Moderate<br>□ Small<br>□ Not a concern, **move to next item**	□ Yes<br>□ No…**Next item**	□ Very easy<br>□ Easy<br>□ Hard<br>□ Very hard<br>□ Didn't get any help
Taking care of children, elders, or other family members	□ Big<br>□ Moderate<br>□ Small<br>□ Not a concern, **move to next item**	□ Yes<br>□ No…**Next item**	□ Very easy<br>□ Easy<br>□ Hard<br>□ Very hard<br>□ Didn't get any help
Difficulty getting health or life insurance	□ Big<br>□ Moderate<br>□ Small<br>□ Not a concern, **move to next item**	□ Yes<br>□ No…**Next item**	□ Very easy<br>□ Easy<br>□ Hard<br>□ Very hard<br>□ Didn't get any help
Paying health care bills (e.g., treatment, services, travel to appointments, assistive devices)	□ Big<br>□ Moderate<br>□ Small<br>□ Not a concern, **move to next item**	□ Yes<br>□ No…**Next item**	□ Very easy<br>□ Easy<br>□ Hard<br>□ Very hard<br>□ Didn't get any help

©2020 Shakeel S et al. *JAMA Network Open.*
